# Egg Spots Are Important Cues for Egg Recognition in Barn Swallows

**DOI:** 10.1002/ece3.71235

**Published:** 2025-04-09

**Authors:** Kui Yan, Jinmei Liu, Kangning Luo, Wei Liang

**Affiliations:** ^1^ Ministry of Education Key Laboratory for Ecology of Tropical Islands, Key Laboratory of Tropical Animal and Plant Ecology of Hainan Province, College of Life Sciences Hainan Normal University Haikou China

**Keywords:** brood parasitism, common cuckoo, egg recognition, egg shape, egg spot

## Abstract

Egg recognition is one of the most common strategies utilized by host birds to combat brood parasitism. Eggshell surface features (e.g., eggshell color and spots) are important cues for host egg recognition, enabling avian hosts to recognize and reject foreign eggs. The barn swallow ( *Hirundo rustica* ), as one host of the common cuckoo ( *Cuculus canorus* ), has demonstrated variation in their egg rejection propensity geographically. However, it remains unclear whether eggshell spots play an important role in their egg recognition. To this end, this study examined the role of eggshell spots in egg recognition in two populations of barn swallows. Our results revealed that in both Danzhou and Dongfang populations, the rejection rate of barn swallows for spotted eggs was significantly lower than that for eggs without spots, suggesting that eggshell spot signaling plays a pivotal role during the process of egg recognition in barn swallows. Our findings also indicated that egg shape (ovoid vs. round) did not affect egg recognition by barn swallows. This was most likely because the round shape did not deviate significantly from egg‐shaped properties, causing the inability of barn swallows to distinguish the difference in shape.

## Graphical abstract

We experimentally tested if egg spots were used as recognition cues in barn swallows ( *Hirundo rustica* ). The rejection rate of barn swallows for spotted eggs was significantly lower than that for spotless eggs, suggesting that eggshell spot signaling plays a pivotal role during the process of egg recognition in barn swallows.

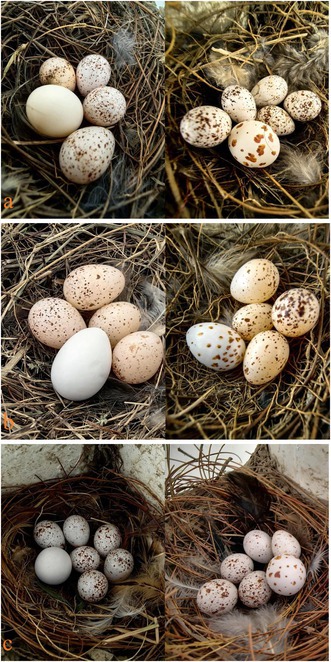

## 1 Introduction

Some birds have evolved egg recognition behaviors in response to selection pressures from dense colonial nesting, nest usurpation, and brood parasitism (Underwood and Sealy 2002). In brood parasitism systems, birds such as cuckoos (*Cuculus* spp.) engage in parasitic behavior by laying their eggs in the nests of host birds, thus successfully transferring the reproductive costs of raising offspring to the host (Rothstein 1990; Davies 2000, 2015). If brood parasitism imposes significant costs, hosts will evolve effective anti‐parasitism strategies, such as repelling or attacking parasites close to the nest to prevent them from laying eggs (nest defense; Li et al. 2015); or recognizing and rejecting foreign eggs or foreign nestlings to prevent successful parasitism (egg or nestling recognition; Langmore et al. 2003; Hanley et al. 2019). Among these, the recognition and rejection of parasitic eggs are the most common defenses against brood parasitism adopted by hosts (Davies 2000; Soler 2014, 2017).

However, parasitic eggs are not always easily recognized by the host. In response to host egg recognition, most parasitic birds have evolved counter‐adaptations, such as laying mimetic or cryptic eggs, to overcome host egg recognition and rejection (Gloag et al. 2014; Abernathy et al. 2017). Currently, at least 60% of cuckoo eggs have been identified to mimic one or more species of host eggs (Payne 2005). In general, cuckoo eggs mimic not only the background color of the host egg (Stoddard and Stevens 2011) but even the spot color, spot distribution, and shape of the host egg (Stoddard and Stevens 2010; Soler 2014). Egg mimicry complicates the detection of parasitic eggs by the host, thereby compelling the host to raise unrelated offspring (Cassey et al. 2008; de la Colina et al. 2012; Stokke et al. 2017). As a result, in response to stronger parasitic pressure, hosts typically evolve more sophisticated egg recognition behaviors to assess the risk of accepting a parasitic egg (acceptance error) or incorrectly rejecting their own egg (rejection error). For example, some avian hosts can use a variety of signals as egg recognition cues to minimize the risk of recognition errors (Spottiswoode and Stevens 2010; Hanley et al. 2019). Numerous studies have found that signals such as egg size, egg shape, egg color, and egg spotting can be involved in host egg recognition and rejection (Underwood and Sealy 2006; Yang et al. 2010; Liu et al. 2019, 2023). However, the relative contributions of different signaling cues to host egg recognition and rejection vary significantly (Honza and Cherry 2017).

Among the many egg recognition signals, egg shape appears to have little effect on host egg recognition (Underwood and Sealy 2006), whereas egg color and egg spots may play a more important role (Feeney et al. 2014). Since variable egg coloration and spot patterns can generate distinct egg characteristics (Davies 2000; Hauber 2014; Stoddard et al. 2014), these features can provide valuable information to the host regarding egg ownership (own vs. foreign; Dainson et al. 2017). For example, López‐de‐Hierro and Moreno‐Rueda (2010) found that egg‐spot pattern was a key factor influencing egg recognition in the house sparrow (*Passer arcticus*; but see Yang et al. 2015). Another example is the study by Liu et al. (2019), which demonstrated that egg spots serve as an important egg recognition cue for cinereous tits ( *Parus cinereus* ). Interestingly, the explanation of egg spots has not been explored for barn swallows ( *Hirundo rustica* ) with large populations and wide distributions.

There are currently four main explanations for the role of egg spots: (1) egg spots are associated with protective coloration, as they enhance egg concealment in open nests (Kilner 2006); (2) egg spots are associated with sexually selective functions, as the pigments from the spots can signal either the quality of the female or the willingness of the female to reproduce (Poláček et al. 2017); (3) egg spots are related to egg structural function, as they can improve eggshell strength (García‐Navas et al. 2011); (4) egg spots are associated with brood parasitism, as they provide unique egg signatures (Davies 2000; Stoddard et al. 2014). We predict egg spots are an important cue for egg recognition in barn swallows，because egg color and egg spot patterns are heritable (Gosler et al. 2000), which would help the host to fully understand the characteristics of their own eggs, and hence facilitate the detection of foreign eggs (Stoddard et al. 2014; Caves et al. 2015).

Populations of barn swallows are abundant and widely distributed in North America, Europe, Asia, and North Africa (Callaghan et al. 2021). In China, a long history of co‐evolutionary interactions has been observed between barn swallows and common cuckoos ( *Cuculus canorus* ; Li et al. 2024), with parasitism rates ranging from 0% to 2.4% (Su et al. 2017; Yang and Feeney 2022). The eggs of barn swallows are usually oval with a white background and dark red spots of varying sizes (Li et al. 2019). In Hainan Island, barn swallows have been shown to possess some degree of egg recognition, with a rejection rate of nearly 50% for non‐mimetic eggs (Liang et al. 2013; Yang et al. 2022). A further study showed that barn swallows rejected more white and blue model eggs with shorter reflectance spectra than red model eggs with longer reflectance spectra (Yan and Liang 2024). However, whether spots play a role in the recognition of barn swallow eggs has not been studied. To this end, we further explored the egg recognition ability of barn swallows in different geographical populations, paying special attention to the role of egg spots in the egg recognition of barn swallows. If egg spot is a key factor influencing egg recognition in the barn swallow, we expect that barn swallows would reject more eggs without spots but fewer spotted eggs. If egg spots do not serve as egg recognition cues, then we expect that there would be no significant difference in egg rejection by barn swallows between spotted eggs and eggs without spots.

## 2 Materials and Methods

### 2.1 Study Area

The study areas are located in Danzhou (19°11′–19°52′ N, 108°56′–109°46′ E) and Dongfang (18°43′ –19°18′ N, 108°36′–109°07′ E) in the western part of Hainan Island. Hainan Island is located in southern China, spanning an area of 33,900 km^2^. It is at the northern edge of the tropics and has a tropical monsoon climate with a wide variety of plants. The average temperature is 22.5°C–25.6°C with an annual precipitation of 1500–2500 mm (Yang et al. 2019). Barn swallows in Hainan Island mostly build nests under the eaves of residential houses, with the breeding period occurring between April and July (Yang et al. 2022). Our study was conducted in scattered villages in Danzhou (March–April, 2020) and Dongfang (March–April, 2021), where we searched for barn swallow nests with eggs along the eaves of residential houses to conduct egg recognition experiments.

### 2.2 Egg Experiments

**FIGURE 1:**
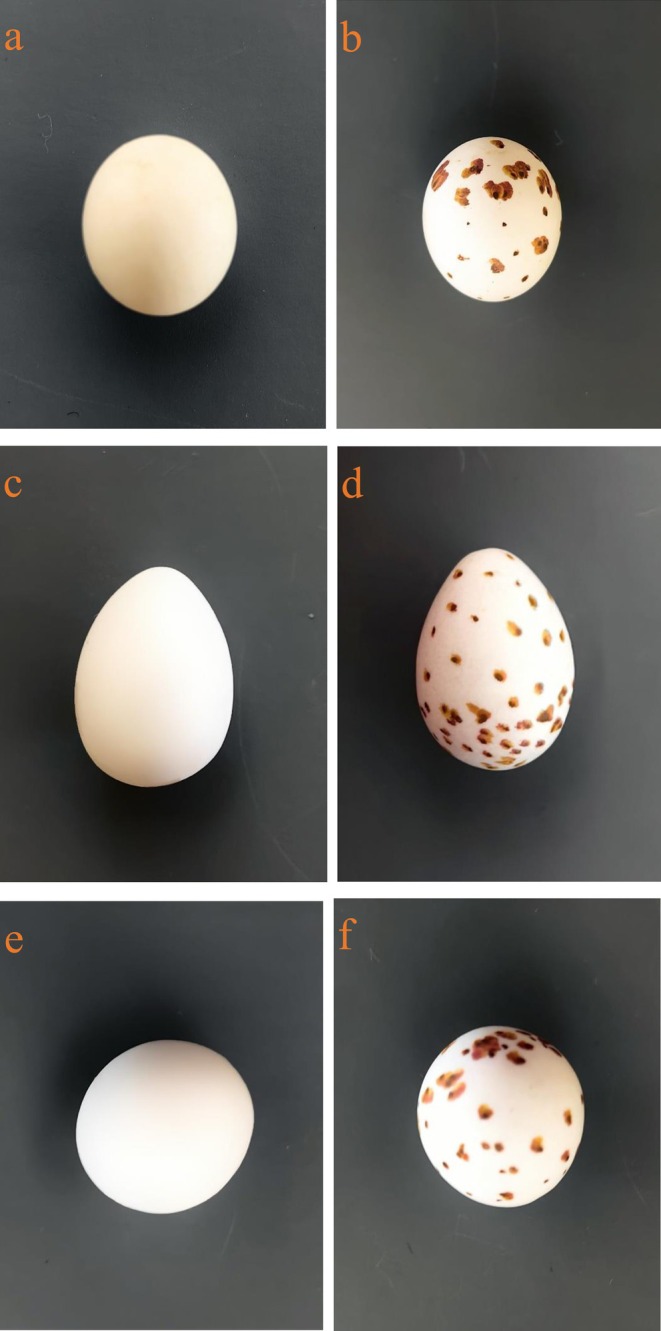
Different experimental eggs (a–f). Letter a refers to the budgerigar ( *Melopsittacus undulatus* ) egg (B‐egg), b refers to spotted budgerigar egg (SB‐egg), c refers to budgerigar egg‐sized white model egg (BM‐egg), d refers to budgerigar egg‐sized spotted white model egg (SBM‐egg), e refers to swallow egg‐sized white round model egg (RM‐egg), and f refers to swallow egg‐sized spotted white round model egg (SRM‐egg), respectively (Photo by Kangning Luo).

**FIGURE 2:**
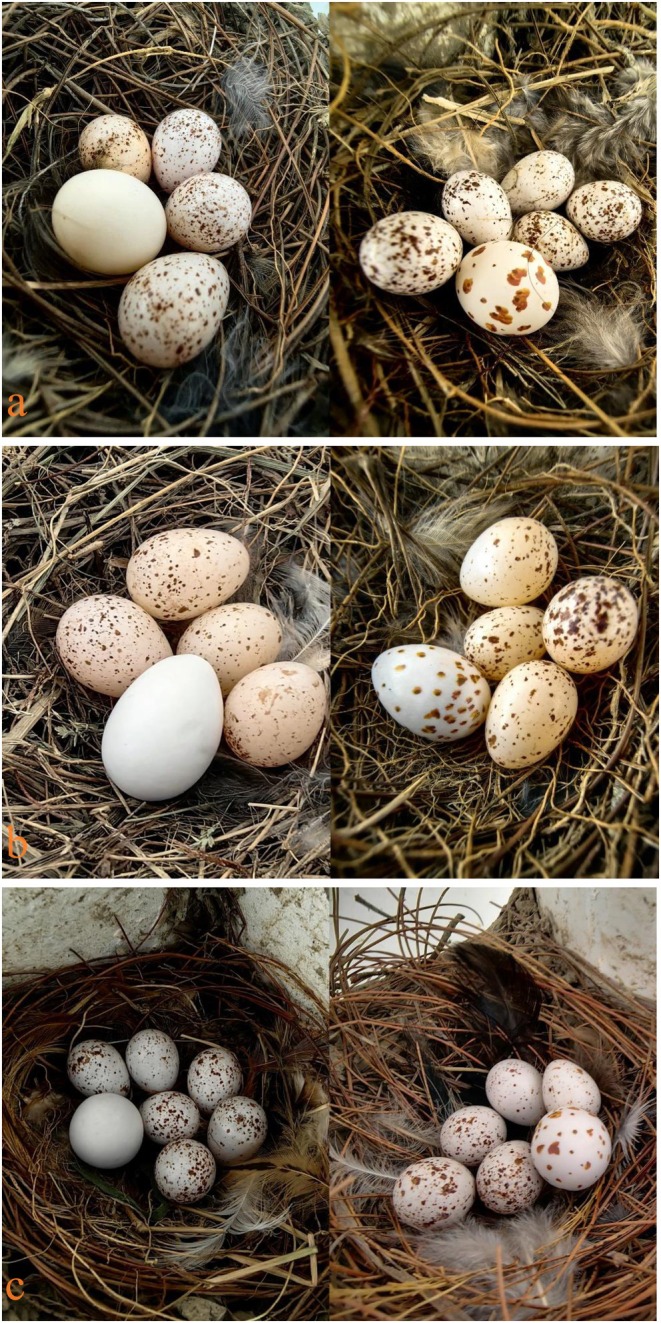
Protocol used for the egg experiments. Images show the addition of B‐egg, SB‐egg, BM‐egg, SBM‐egg, RM‐egg, and SRM‐egg to the barn swallow nest, respectively (photo by Kangning Luo).

**FIGURE 3:**
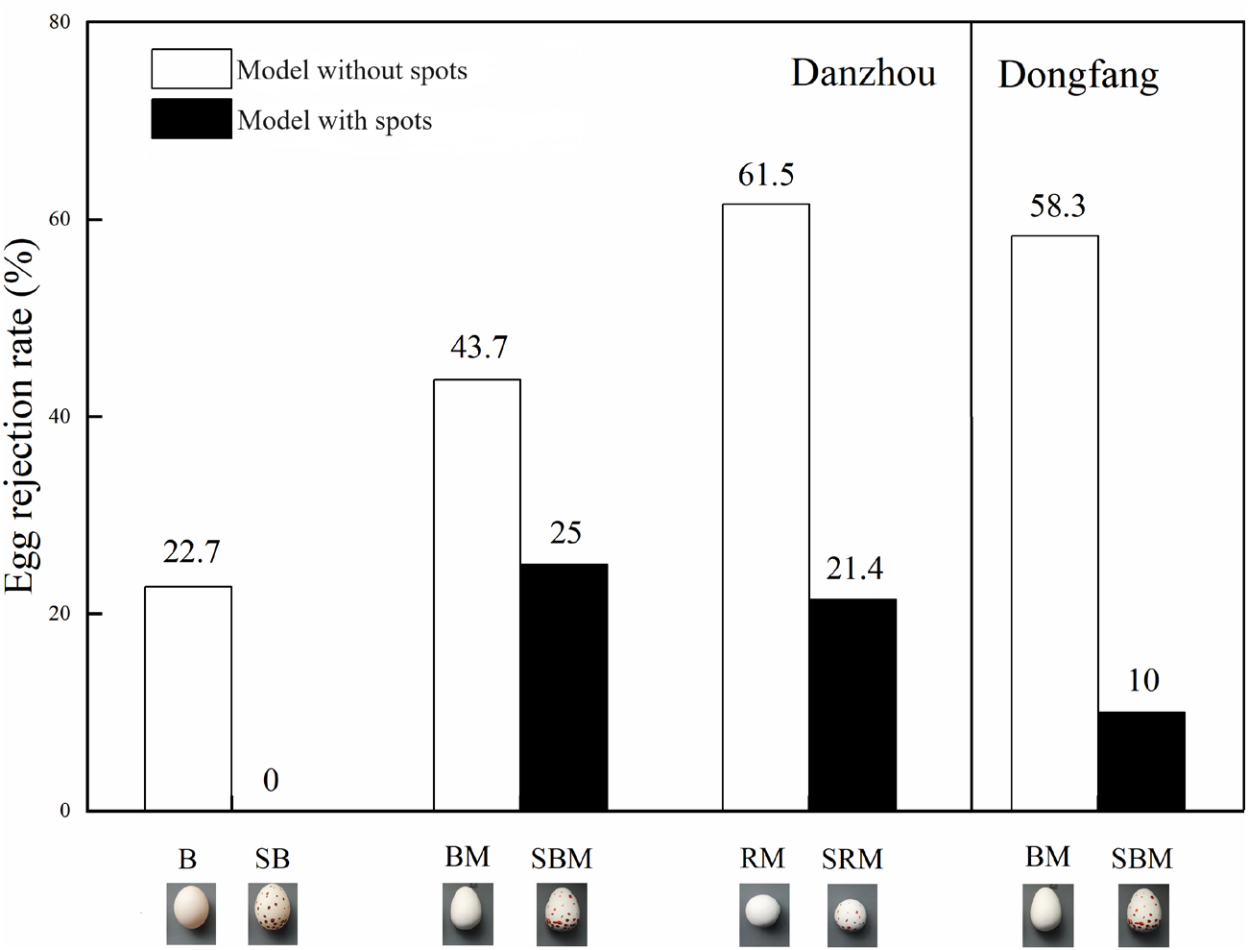
Egg rejection rates of different experimental eggs in barn swallow populations of Danzhou and Dongfang populations, Hainan, China. The number on the bar refers to egg rejection rate of experimental eggs.

Two weeks before the start of the egg experiment in the barn swallow nest with eggs, we prepared the different types of experimental eggs needed. Pure white budgerigar ( *Melopsittacus undulatus* ) eggs (slightly larger than barn swallow eggs) were purchased from the Taobao website (hereafter B‐egg, Figure 1a), which were marked with spots (hereafter SB‐egg, Figure 1b) using dark red professional marker pens (APM 25201, M & G). Four types of model eggs were made using white polymer clay (Ai Tao Le, Shenzhen, China) and professional marker pens: (1) budgerigar egg‐sized white model eggs (hereafter BM‐egg, Figure 1c); (2) budgerigar egg‐sized white model eggs marked with dark red spots (hereafter SBM‐egg, Figure 1d); (3) white round eggs with a diameter equivalent to the width of barn swallow eggs (hereafter RM‐egg, Figure 1e); (4) white round eggs with a diameter equivalent to the width of barn swallow eggs and marked with dark red spots (hereafter SRM‐egg, Figure 1f).

We systematically searched for barn swallow nests in villages in Danzhou in late February 2020, the period when barn swallows began to arrive and breed (Yang et al. 2022). Active nests were checked regularly (approximately every 3 days), and when egg laying was detected, the nests were monitored daily, recording clutch size and the time when eggs hatch. During the early stage of egg incubation (≤ 4 days of egg incubation), one B‐egg (*n* = 22), SB‐egg (*n* = 19), BM‐egg (*n* = 16), SBM‐egg (*n* = 16), RM‐egg (*n* = 13), or SRM‐egg (*n* = 14) was added to the nests (Figure 2). Similarly, to test the effect of egg spots on egg recognition in different barn swallow populations (villages in Dongfang), egg‐spot recognition experiments were conducted during the same period in 2021, where one BM‐egg (*n* = 12) or SBM‐egg (*n* = 10) was added to the nests of barn swallows in the early stage of egg incubation. Only one type of egg experiment was performed on each barn swallow nest, and each egg experiment lasted for 6 days (Yang et al. 2022). The nests were checked on day 2 and day 6 of the experiment to determine if the experimental eggs were rejected. To avoid pseudoreplication of sampled nests from the same barn swallow parents, we chose to complete all egg experimental manipulations within 28 consecutive days, as the breeding cycle of barn swallows (from the start of egg incubation to fledging) lasts approximately 38 days (Yang et al. 2022). If the foreign eggs were removed, buried, or had obvious peck marks during the experimental period, then the barn swallows were considered to have rejected the experimental eggs. Conversely, if the experimental eggs were still intact in the active nest, the barn swallows were considered to have accepted them.

### 2.3 Statistical Analyses

Statistical analysis was performed using IBM SPSS 22.0 software for Windows (IBM Corp., Armonk, NY, USA). For the egg manipulation experiments performed in the Danzhou population, a generalized linear mixed model (GLMM) was used to analyze the differences in egg rejection rates between different experiments. In this model, the egg rejection rate was the response variable; clutch size, type of foreign egg (i.e., B‐egg, BM‐egg, RM‐egg), and egg spots (spotted or without spots) were the fixed variables; and nest ID was the subject. Similarly, the GLMM was performed to analyze the differences in the egg rejection rates between different egg manipulation experiments in the Dongfang population. In this model, the egg rejection rate was the response variable; clutch size and egg spots (spotted or without spots) were the fixed variables; and nest ID was the subject. *p* < 0.05 indicated statistical significance.

## 3 Results

Our results showed that clutch size did not affect the rejection rate of foreign eggs by barn swallows in either the Danzhou or Dongfang population (GLMM, *p* = 0.682; *p* = 0.167, Table 1). However, the rejection rate of barn swallows for spotted eggs (SBM) was significantly lower than that for eggs without spots (BM) in Dongfang (GLMM, *p* = 0.049, Table 1; egg rejection rates of different experimental eggs are shown in Figure 3), and the rejection rate of barn swallows for spotted eggs (SB, SBM and SRM) was significantly lower than that for eggs without spots (B, BM and RM) in Danzhou (GLMM, *p* = 0.023, Table 1; egg rejection rates of different experimental eggs are shown in Figure 3). In addition, our results showed that the rejection rate of barn swallows for round model eggs (RM and SRM) was higher than that for budgerigar egg‐sized model eggs (BM and SBM) and budgerigar eggs (B and SB; Figure 3). However, no significant difference was observed in the rejection rates for the three different types of foreign eggs (GLMM, *p* = 0.073, Table 1).

**TABLE 1:** Generalized linear mixed model (GLMM) analysis of differences in egg rejection rates among different populations of barn swallows.

	*F*	df_1_	df_2_	*p*
Danzhou population				
Clutch size	0.175	1	15	0.682
Egg type	3.138	2	15	0.073
Egg spot	6.381	1	15	0.023
Dongfang population				
Clutch size	2.065	1	19	0.167
Egg spot	4.442	1	19	0.049

*Note:* Clutch size, egg type (ovoid or round), and egg spot (with or without) are fixed factors.

## 4 Discussion

The results of our egg experiments revealed that the rejection rate of barn swallows for spotted eggs was significantly lower than that for eggs without spots in both Danzhou and Dongfang populations. These results suggest that egg spots play a vital role in the process of egg recognition and egg rejection by barn swallows. Furthermore, no differences in the rejection rate of barn swallows were observed for different types of foreign eggs (round vs. ovoid models), which implies that egg shape is not a major cue for egg recognition in barn swallows.

Barn swallow eggs have a white background with dark red spots of varying sizes (Li et al. 2019). When we simulated dark red spots on white eggs, barn swallows showed a significantly lower rejection rate for spotted white eggs than for white eggs without spots, suggesting that eggshell spots play a pivotal role in egg recognition by barn swallows. Swynnerton (1918) previously suggested that eggshell spotting is a feature employed by hosts as an anti‐parasitism strategy, primarily against the mimicry of host eggs by cuckoos. Our findings demonstrated support for the claim made by Swynnerton. Several studies have also found that egg spotting is an important egg recognition cue. For example, Underwood and Sealy (2006) explored the egg features used by warbling vireos ( *Vireo gilvus* ) to distinguish the parasitic eggs of the brown‐headed cowbird ( *Molothrus ater* ) and found that only egg‐spot patterns significantly affected the probability of rejecting foreign eggs by warbling vireos, whereas size and nesting stage had no effect. López‐de‐Hierro and Moreno‐Rueda (2010) found that egg recognition in the house sparrow was not affected by changes in egg size, whereas changes in spot patterns emerged as a key factor affecting egg recognition in this species (but see Yang et al. 2015). Liu et al. (2019) found in cinereous tits (now as Japanese tits *Parus minor hainanus*) that eggshell spots fulfilled a signaling function that may be critical for recognizing and rejecting parasitic eggs.

There are numerous different signals that can influence the host's egg recognition response, including the background color, spotting, shape, and size of the host eggs. Moreover, the host may use the most effective cues or may combine several different egg characteristics to make a rejection decision (Rothstein 1982; Spottiswoode and Stevens 2010). A large body of research has been conducted on the role of egg spots in the recognition of foreign eggs, with the main viewpoints summarized as follows: (1) egg spots contribute little to host egg recognition, and egg size is the main cue for egg recognition (Marchetti 2000; Langmore et al. 2003), and egg color is more important than spot patterns (Lahti and Lahti 2002; Luro et al. 2018); (2) egg spots contribute significantly to host egg recognition, with spot pattern and egg color considered more important than egg size (Rothstein 1978; Igic et al. 2015), and egg spots are considered more important than egg color (Lawes and Kirk‐man 1996; Underwood and Sealy 2006; López‐De‐Hierro and Moreno‐Rueda 2010; de la Colina et al. 2012). Our results support the second viewpoint that egg spots are an important cue for egg recognition in barn swallows, with egg spots playing a more important role than egg size.

Eggshell markings are regarded as the fingerprints of individual females (López‐de‐Hierro and Moreno‐Rueda 2010); additionally, the blunt pole of the egg usually contains more spotting information and serves an important signal for the recognition of parasitic eggs (i.e., the “blunt egg pole” hypothesis; Polačiková et al. 2007, 2010; Polačiková and Grim 2010). A recent study found that the yellow‐bellied prinia ( *Prinia flaviventris* ) exhibited a higher rejection rate for eggs marked with spots on the blunt pole than on the sharp pole (Wang et al. 2020). Overall, our experiments clearly demonstrated that egg spots played a key role in egg recognition among barn swallow populations. However, other potential cues, e.g., egg coloration, color contrast, and position of patterning (Di Giovanni et al. 2023), could not be excluded as our experimental design did not control for these factors. In addition, we did not explore the effect of differences in spot location on egg recognition by barn swallows, which should be explored in future studies.

Parent birds require cognitive abilities to distinguish their own eggs from other objects (Rothstein 1975; Peer 2017; Di Giovanni et al. 2023). Previous studies have used models with different shapes to study host egg recognition behavior (Yang, Wang, et al. 2015; Yang, Chen, et al. 2015; Peer 2017; Luro and Hauber 2017). The ability of parent birds to recognize objects other than eggs depends on the type and shape of the object (Stoddard et al. 2017). Yang et al. (2019) showed that barn swallows were able to distinguish between non‐egg‐shaped and egg‐shaped objects through surface edges and three‐dimensional structure. They found that the cognitive capacity increased as the objects deviated from egg‐shaped to non‐egg‐shaped properties. These findings suggest that the greater the deviation of the objects from egg‐shaped properties, the more frequently the birds recognized these objects. However, our results showed that the shape of foreign objects (round vs. ovoid) did not significantly affect egg rejection. This may be attributed to the preference of swallows for using the presence of spots as a cue for recognizing foreign eggs while disregarding the differences in egg shapes. This could also be due to the fact that round objects do not deviate significantly from egg‐shaped properties, resulting in an inability to recognize differences in shape.

In conclusion, our results are in line with other studies where spots reduce the likelihood of egg rejection and support the hypothesis that spots on the eggshell are a crucial cue in egg recognition in barn swallows. However, egg spot color and the specific features of spot patterns, including spot distribution, size, and brightness, may also serve as potential cues for hosts (Di Giovanni et al. 2023). This study provides the foundation for future research to investigate these spot characteristics and determine which of them are used for egg recognition and rejection behavior in cuckoo hosts.

## Author Contributions

**Kui Yan:** investigation (lead), methodology (lead), resources (lead), writing – original draft (equal). **Jinmei Liu:** formal analysis (lead), validation (equal). **Kangning Luo:** investigation (equal), visualization (equal). **Wei Liang:** conceptualization (lead), funding acquisition (lead), supervision (lead), validation (equal), writing – review and editing (equal).

## Ethics Statement

The experiments comply with the current laws of China, where they were performed. Experimental procedures were in agreement with the Animal Research Ethics Committee of Hainan Provincial Education Centre for Ecology and Environment, Hainan Normal University (no. HNECEE‐2012‐001).

## Conflicts of Interest

The authors declare no conflicts of interest.

## Data Availability

Data used for this study are provided as Supporting Information (Data table S1 and Data table S2) and can be found at https://figshare.com/s/25d8e8fb697869c99a00 (doi: 10.6084/m9.figshare.28122368).
